# Solvent-Mediated Control of Twisted Intramolecular Charge Transfer in 7-(Diethylamino)coumarin-3-carboxylic Acid

**DOI:** 10.3390/molecules31010076

**Published:** 2025-12-24

**Authors:** Xilin Bai, Jing Xiao, Bingqi Du, Duidui Liu, Yanzhuo Wang, Shujing Shi, Jing Ge

**Affiliations:** 1 School of Physics and Electronic Engineering, Shanxi Normal University, No. 339, Taiyu Road, Taiyuan 030031, China; 2 Department of Chemical Physics, University of Science and Technology of China, No. 96, Jinzhai Road, Hefei 230026, China

**Keywords:** 7-(diethylamino)coumarin-3-carboxylic acid, solvent polarity, hydrogen bonding, twisted intramolecular charge transfer, femtosecond transient absorption

## Abstract

Understanding the influence of solvent environments on the excited-state charge transfer process remains a fundamental question in molecular photophysics and photochemistry. While twisted intramolecular charge transfer (TICT) is crucial in determining fluorescence efficiency and photostability, the combined effects of solvent polarity and hydrogen bonding interactions are still elusive. Here, we employ steady-state and femtosecond transient absorption (fs-TA) spectroscopy with density functional theory (DFT) calculations to investigate the excited-state dynamics of 7-(diethylamino)coumarin-3-carboxylic acid (7-DCCA) in different solvents. Our findings reveal that in highly polar solvents with strong hydrogen-donating and hydrogen-accepting capabilities, 7-DCCA undergoes significant TICT formation, resulting in fluorescence quenching. Conversely, in environments with low polarity or weak hydrogen-bonding interactions, this transformation is largely suppressed. Quantitative correlation analysis utilizing the Kamlet–Taft and Catalán four-parameter models further elucidates the synergistic role of solvent polarity and specific hydrogen-bonding parameters in modulating the steady-state spectral behavior of 7-DCCA. This study provides microscopic insights into solvent–charge transfer interactions and establishes a general framework for enhancing the luminescence efficiency and structural robustness of organic optoelectronic materials through strategic solvent engineering.

## 1. Introduction

Twisted intramolecular charge transfer (TICT) is one of the most characteristic excited-state processes, playing a critical role in determining fluorescence efficiency, excited-state stability, and the performance of optoelectronic materials. In conventional donor-acceptor (D-A) molecules, photoexcitation induces an intramolecular charge transfer (ICT) process; upon conformational twisting between the donor and acceptor units, a highly polarized TICT state can emerge [1,2,3,4,5]. Therefore, achieving a controllable balance between ICT and TICT is central to the rational design of high-performance luminescent molecules, fluorescent probes, and optoelectronic materials. Despite decades of research, a comprehensive microscopic understanding of how solvent environments regulate the TICT process remains elusive.

Pioneering studies by Lippert et al. revealed the profound influence of solvent polarity on ICT emission through dual fluorescence observations in 4-(*N*,*N*-dimethylamino) benzenecarbonitrile (DMABN) [6,7,8,9]. Building upon this foundation, Grabowski et al. proposed the TICT model, identifying the twisting of the Me_2_N group relative to the phenyl group as the defining conformational feature of the TICT state [10,11,12,13]. Subsequent research has established that both solvent polarity and hydrogen bonding substantially reshape the excited-state potential energy landscape of TICT-active molecules [14,15,16,17,18]. However, the cooperative or competitive relationship between these two factors in determining excited-state dynamics and fluorescence characteristics remains poorly understood, representing a long-standing challenge in molecular photophysics and photochemistry.

**Figure 1:**
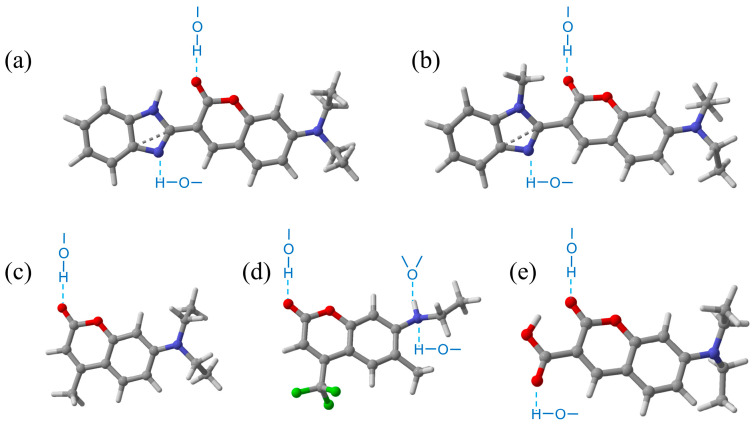
The structural diagrams of C7 (**a**), C30 (**b**), C1 (**c**), C307 (**d**) and 7-DCCA (**e**).

Coumarin-based dyes, with their well-defined D-A architectures and tunable optical properties, have become ideal model systems for studying the TICT process. Li et al. revealed the significant impact of hydrogen bonding on coumarin 1 (C1), showing that C1 can form a TICT state perpendicular to the aromatic ring in methanol via DFT/TDDFT calculations [19]. Ge et al. discovered that high-polarity solvents and enhanced intermolecular hydrogen bonds can stabilize the TICT state of coumarin 307 (C307) [20], while recent studies have shown that coumarin 30 (C30) exhibits significant TICT behavior in polar solvents (such as MeOH and ACN), while coumarin 7 (C7) maintains a planar structure due to the stabilizing effect of intramolecular hydrogen bonds, thereby effectively inhibiting the occurrence of TICT [21]. The structures of these coumarin derivatives are shown in Figure 1, where the light blue dashed lines represent the positions where hydrogen bonds are formed between the solvent and solute molecules.

7-(Diethylamino)coumarin-3-carboxylic acid (7-DCCA), a typical amino-coumarin dye (as shown in Figure 1e), with a high fluorescence quantum yield and a well-defined D-A configuration, is an ideal model molecule for investigating solvent effects. Its photophysical properties are highly sensitive to solvent polarity and hydrogen-bonding ability, implying that the synergistic interaction between these two factors governs the population distribution between the locally excited (LE) and TICT states [22,23,24]. However, there is still a lack of systematic research combining experiments and theory to understand the mechanism by which the polarity of the solvent and hydrogen bond interactions jointly regulate the formation of TICT at the energy and conformational levels.

Here, we combine steady-state absorption and fluorescence with femtosecond transient absorption spectroscopy experiments, supported by density functional theory (DFT) and time-dependent density functional theory (TDDFT) calculations, to unravel the excited-state dynamics of 7-DCCA. Further, by applying the Kamlet-Taft (K-T) and Catalán four-parameter (4P) models [25,26,27,28,29], the intrinsic relationship between solvent properties and spectral responses is quantitatively analyzed. The results establish a unified mechanistic framework for solvent-induced TICT state formation, providing novel physical insights and theoretical guidance for the molecular design of efficient and stable fluorescent materials.

## 2. Results and Discussion

### 2.1. Kamlet-Taft Model and Catalán 4P Model

The effects of solvent properties on 7-DCCA were analyzed based on the K-T parameters (*α*, *β*, π*) [30,31] and the Catalán 4P (*SA*, *SB*, *SP*, *SdP*) [26]. In the K-T model, *α* represents the hydrogen-donating ability, *β* represents the hydrogen-accepting ability, and π* denotes the solvent polarity. In the Catalán model, *SA* and *SB* correspond to solvent acidity and basicity, and *SP* and *SdP* describe solvent polarizability and dipolarity. In this study, solvents from Table 1 were selected to investigate these two models. The corresponding steady-state absorption and fluorescence emission wavelengths are shown in Table 2.

To explore the dependence of solvent polarity and hydrogen bonding ability on the steady-state spectral parameters of 7-DCCA, we conducted an analysis using the Kamlet-Taft method. This method provides a detailed explanation of the distinction between the effects of solvent polarity and hydrogen bonding ability [32]. A quantitative analysis of solvent effects on the steady-state spectral characteristics of 7-DCCA was performed using the Linear Solvation Energy Relationship (LSER) approach, expressed as:

(1) $$v-v_{0}=a\alpha +b\beta +s\pi^{*}\;$$

where v represents the spectral peak frequency of the solute in a given solvent, and $v_{0}$ corresponds to the peak frequency of the solute in the gas phase. Additionally, we performed an analysis using the Catalán 4P model, as shown in Equation (2). In solvatochromic analysis, solvent effects are divided into specific and non-specific interactions. When the solute is non-polar or weakly polar, the Kamlet-Taft model performs poorly. Non-specific interactions are represented using a new scaling of the Catalán solvent polarity parameter, which is further modified to include solvent polarizability (*SP*) and solvent dipolarity parameter (*SdP*) to distinguish the differences from the Kamlet-Taft model, which mixes specific and non-specific effects in the solvent [31]. This model approximates the solute-solvent interactions using a dielectric continuum:

(2) $$v=v_{0}+C_{SA}SA+C_{SB}SB+C_{SP}SP+C_{SdP}SdP$$

where the coefficients $C_{SA}$, $C_{SB}$, $C_{SP}$ and $C_{SdP}$ represent regression coefficients obtained from multivariate linear fitting. The data from Table 1 and Table 2 were used to fit these two models, and the results are shown in Table 3. In the K-T model, a larger fitting coefficient s indicates that the solvent polarity has a more significant impact on the solute’s spectral frequency, meaning that the absorption or emission spectra of 7-DCCA will change more with increasing solvent polarity. If s is positive, the solute’s spectrum may experience a red shift; if negative, a blue shift may occur. The coefficients a and b quantify the effects of hydrogen bond donor and acceptor abilities on the solute’s spectrum. Solvents with strong hydrogen bond donor or acceptor abilities can form intermolecular hydrogen bonds with the solute, altering its electronic structure. Conversely, if the value is negative, it indicates that these solvents have a weaker stabilizing effect on the solute’s excited state, potentially leading to instability in the electronic structure. Similarly, the impact of each coefficient in the Catalán 4P model on the 7-DCCA spectrum can be analyzed. As summarized in Table 3, hydrogen-donating ability, hydrogen-accepting ability, and polarizability are key factors influencing the absorption, fluorescence, and Stokes shifts, with the solvent polarizability having the most significant effect.

### 2.2. Steady-State Absorption and Fluorescence Spectra

**Figure 2:**
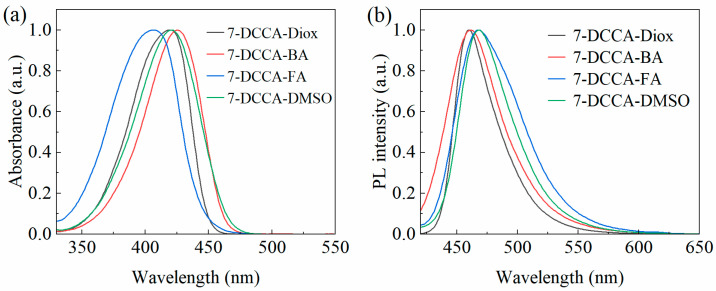
Normalized steady-state absorption (**a**) and fluorescence (**b**) spectra of 7-DCCA in Diox, BA, FA, and DMSO.

We selected four representative solvents—dioxane (Diox), n-butanol (BA), formamide (FA), and dimethyl sulfoxide (DMSO)—from Table 2 for both steady-state and transient spectral analysis, as well as for quantitative analysis. The normalized steady-state absorption and fluorescence spectra of 7-DCCA in Diox, BA, FA, and DMSO are shown in Figure 2. The maximum absorption peaks of 7-DCCA in these solvents occur at 420 nm (Diox), 424 nm (BA), 407 nm (FA), and 422 nm (DMSO), while the maximum emission peaks are at 460 nm (Diox), 461 nm (BA), 470 nm (FA), and 469 nm (DMSO). We observed that the emission band of 7-DCCA exhibits a red shift with increasing solvent polarity, indicating the potential presence of ICT in the excited state [33]. Notably, the Stokes shift differs significantly across solvents: 2017 cm^−1^ (Diox), 1893 cm^−1^ (BA), 3293 cm^−1^ (FA), and 2375 cm^−1^ (DMSO). From Table 1, it can be observed that BA, with strong hydrogen-donating and hydrogen-accepting abilities but low polarity, results in the smallest Stokes shift for 7-DCCA. In contrast, FA, with higher polarity and hydrogen-donating ability, forms stable intermolecular hydrogen bonds with 7-DCCA, effectively promoting charge transfer and stabilizing the ICT state, thereby producing the largest Stokes shift. These results indicate that the synergistic effects of solvent polarity and hydrogen bonding interactions collaboratively regulate the excited state behavior of 7-DCCA.

To further elucidate the electronic nature of these transitions, TDDFT calculations were carried out to determine the vertical excitation energies and oscillator strengths associated with the S_0_ → S_1_ transition in Diox, BA, FA, and DMSO (Table 4). The calculation results indicate that the oscillator strengths corresponding to the S_1_ state of the hydrogen-bonded complexes 7DCCA-Diox, 7DCCA-BA, 7DCCA-FA, and 7DCCA-DMSO are the largest, suggesting that their absorption maxima are located in the S_1_ state, at 405 nm, 412 nm, 403 nm, and 407 nm, respectively. These calculated absorption wavelengths show reasonable correspondence with experimental values (420 nm, 424 nm, 407 nm, and 422 nm, respectively). Similarly, the calculated emission energies are qualitatively consistent with experimental trends (Table 5). This level of agreement is adequate for the interpretative scope of this work, where computational results are used to support mechanistic understanding rather than provide quantitative predictions [34,35]. While B3LYP-based TDDFT is not state-of-the-art for quantitatively describing charge-transfer states in polar environments, it successfully reproduces the solvent-dependent trends observed experimentally. The computational results are therefore used as interpretative tools to support our experimental findings, not as predictive benchmarks.

### 2.3. Transient Absorption Spectroscopy

**Figure 3:**
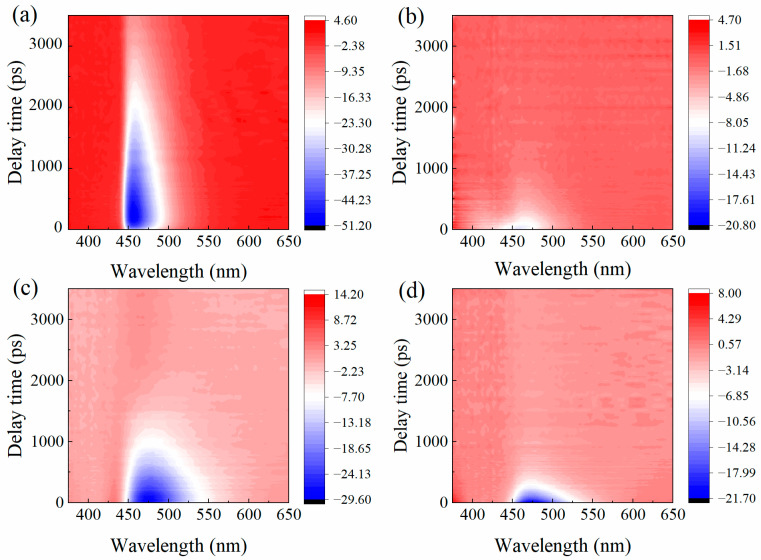
Two-dimensional transient absorption spectra of 7-DCCA in Diox (**a**), BA (**b**), FA (**c**), and DMSO (**d**).

Femtosecond transient absorption (fs-TA) spectroscopy is a powerful technique that enables real-time observation of ultrafast excited-state dynamics induced by laser excitation. Here, fs-TA measurements were employed to elucidate how solvent polarity and hydrogen-bonding interactions modulate the relaxation dynamics of 7-DCCA. To illustrate the spectral evolution over time, two-dimensional transient absorption spectra of 7-DCCA in different solvents were constructed (Figure 3). In these plots, positive signals (red) correspond to excited-state absorption (ESA), while negative signals (blue) denote ground-state bleaching or stimulated emission (SE). The horizontal axis represents probe wavelength (375–650 nm), and the vertical axis corresponds to the delay time (0–3500 ps). From the spectra, it is clearly observed that in the low-polarity solvents Diox and BA, a pronounced negative signal appears around 460 nm, corresponding to an SE band between 430 and 480 nm. In contrast, in high-polarity solvents FA and DMSO, the negative region progressively extends toward longer wavelengths, reaching ~540 nm. This redshift is consistent with the steady-state emission results and indicates that the excited-state relaxation involves the formation of a TICT state.

**Figure 4:**
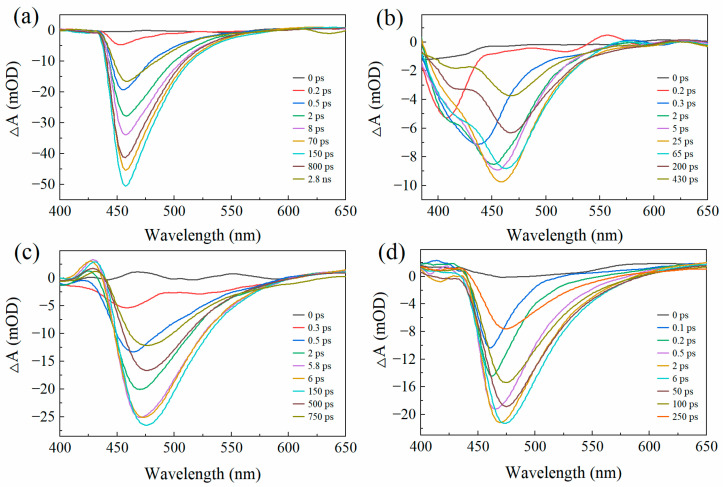
Fs-TA spectra of 7-DCCA in Diox (**a**), BA (**b**), FA (**c**), and DMSO (**d**).

To further probe the solvent dependence of these processes, fs-TA spectra were analyzed at representative delay times (Figure 4). The fs-TA spectra of 7-DCCA hydrogen-bonded complexes under 375 nm excitation exhibit notable solvent dependence. In the low-polarity solvent Diox, the negative band around 456 nm aligns well with the steady-state fluorescence peak at 461 nm, confirming its assignment to SE. In BA, the negative signal extends from 390 to 570 nm and stabilizes around 460 nm after approximately 25 ps. The significant broadening of the emission band is closely related to BA’s strong hydrogen-donating and hydrogen-accepting capabilities, indicating that hydrogen bonding interactions facilitate the ICT process. For high-polarity solvents such as FA and DMSO, the negative signal range extends from 430 to 600 nm, peaking around 475 nm. During the 5.8–6 ps period, the SE peak of the FA system redshifts from 469 nm to 473 nm; similarly, in the DMSO system, the SE peak redshifts from 468 nm to 474 nm over the 2–6 ps period. This time-dependent redshift reflects the structural relaxation process from the ICT state to the TICT state. Overall, the results indicate that high polarity and strong hydrogen-bonding environments jointly enhance the charge transfer characteristics of the S_1_ state and stabilize the TICT configuration, revealing the synergistic regulatory mechanism of solvent polarity and hydrogen bonding in the TICT process [36].

**Figure 5:**
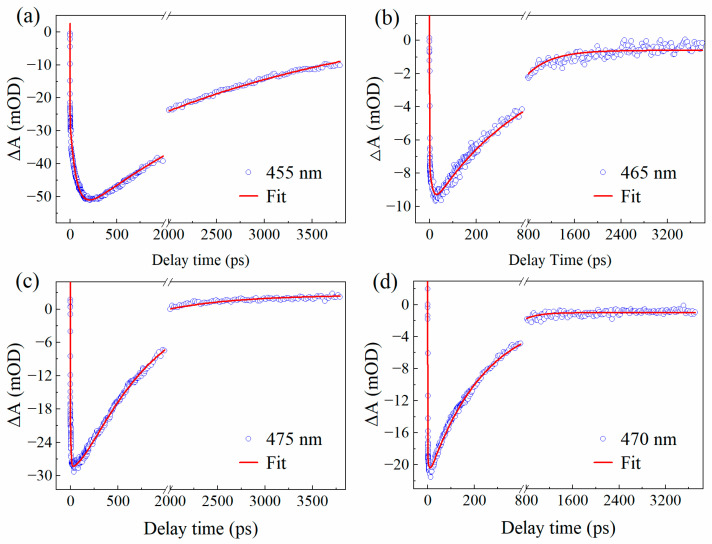
TA kinetic curves of 7-DCCA in Diox (**a**), BA (**b**), FA (**c**), and DMSO (**d**).

To quantify these relaxation dynamics, kinetic traces were extracted at characteristic wavelengths (Figure 5). The kinetic curve of 7-DCCA-Diox at 455 nm increased rapidly within 1 ps, indicating that most of the 7-DCCA molecules in the ground state quickly transitioned to the transient ICT state. A similar phenomenon was observed in the other three hydrogen-bonded complexes. However, in highly polar solvents such as FA and DMSO, the emergence of an additional intermediate state leads to a broadening and a decrease in the SE band, shifting its energy from 430–550 nm to 430–600 nm. Based on subsequent quantitative analysis, we attribute this intermediate state to the TICT state [34]. The kinetic traces at characteristic wavelengths were fitted (parameters summarized in Table 6). In Diox, the decay kinetics fit well to a tri-exponential model. The fastest component, τ_1_ ≈ 0.43 ps, corresponds to the ultrafast transition from the LE state to the ICT state. The subsequent components, τ_3_ ≈ 69.8 ps and τ_4_ ≈ 2.8 ns, are attributed to solvation and radiative decay, respectively. A similar kinetic behavior is observed in BA. However, in the high-polarity solvents FA and DMSO, an additional intermediate component is required for satisfactory fitting, yielding a four-exponential decay. Specifically, the τ_2_ component (~5.51 ps in FA, ~3.01 ps in DMSO) is attributed to the transition from the ICT state to the TICT state. This newly observed intermediate time-scale relaxation reflects the more complex evolution of the excited-state dynamics in high-polarity and strong hydrogen-bonding environments. Overall, the fs-TA results clearly demonstrate that both solvent polarity and hydrogen bonding play pivotal roles in controlling the excited-state relaxation of 7-DCCA. Low-polarity environments favor conventional ICT emission, whereas high-polarity and strongly hydrogen-bonding solvents promote efficient TICT formation and stabilization. These findings provide mechanistic insight into how solvent–solute interactions govern the charge-transfer dynamics of coumarin derivatives, offering a broader understanding of solvent-mediated excited-state processes in donor–acceptor systems.

### 2.4. Theoretical Calculations

**Figure 6:**
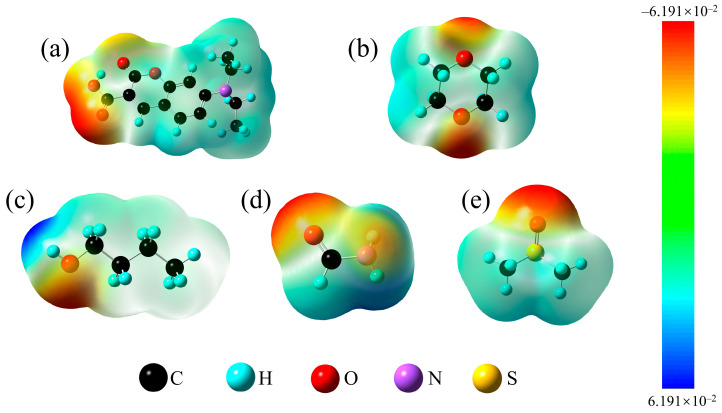
Electrostatic potential maps of 7-DCCA (**a**), Diox (**b**), BA (**c**), FA (**d**), and DMSO (**e**).

To validate the experimental findings, DFT was used to optimize the ground-state frequency and structure of the 7-DCCA hydrogen-bonded complex. Based on this, TDDFT calculations were performed to obtain the corresponding vertical excitation energy. Subsequently, TDDFT was used to optimize the excited-state structure to obtain the fluorescence emission energy. These calculations were carried out at the B3YLP/Def2TZVP level. Additionally, to avoid the occurrence of artificial TICT states, which are commonly observed when scanning the excited-state potential energy surface with B3LYP, a potential energy surface analysis of the 7-DCCA complex was performed at the CAM-B3LYP/Def2TZVP level. All calculations considered D3BJ dispersion correction and the SMD implicit solvent model. Figure 6 presents the electrostatic potential (ESP) maps of 7-DCCA and various solvents. ESP analysis offers a direct visualization of potential hydrogen-bonding interactions between solute and solvent molecules, revealing both the feasibility and specific sites of hydrogen-bond formation [19]. The color scale ranges from red (regions of positive, nucleophilic) to blue (negative potential, electrophilic) [37,38]. In 7-DCCA, the carbonyl (C=O) site exhibits a pronounced red region, indicating strong nucleophilicity and a high propensity to act as a hydrogen-bond acceptor. Conversely, the O–H and C-H sites in solvent molecules appear blue, highlighting their potential as hydrogen-bond donors [39]. In addition, we also calculated the electrostatic potential maps of 7-DCCA in the S_0_ and S_1_ states in different solvents (as shown in Figure S1). From Figure S1, the binding sites of intermolecular hydrogen bonds can also be clearly observed. These complementary electrostatic interactions facilitate the formation of stable intermolecular hydrogen bonds, consistent with the experimental solvent–solute interaction trends.

**Figure 7:**
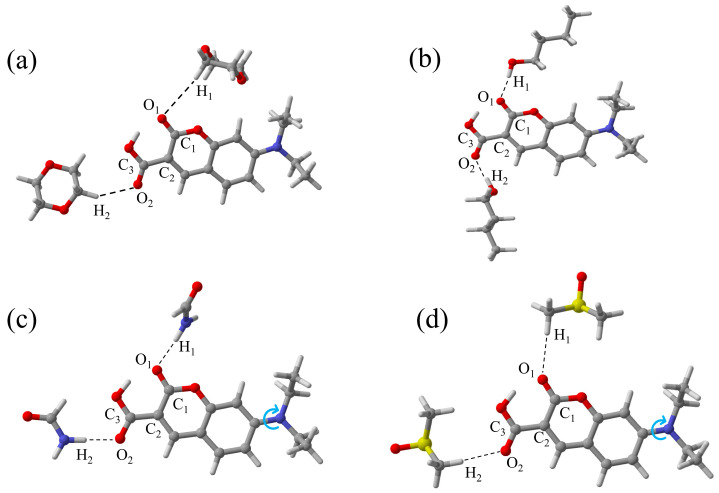
Optimized S_0_ state geometries of 7-DCCA in Diox (**a**), BA (**b**), FA (**c**), and DMSO (**d**).

**Figure 8:**
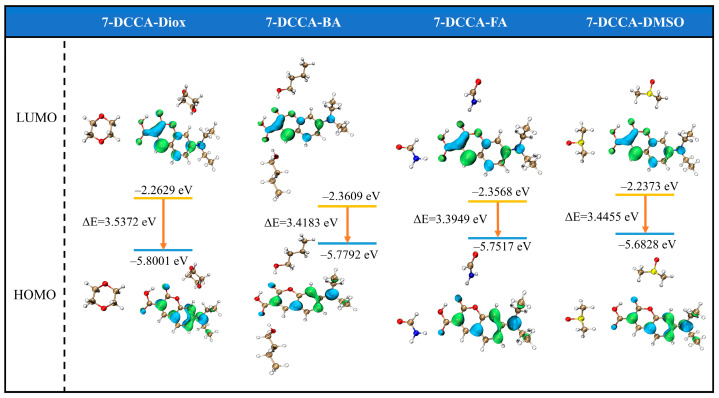
Frontier molecular orbitals of 7-DCCA hydrogen-bonded complexes.

**Figure 9:**
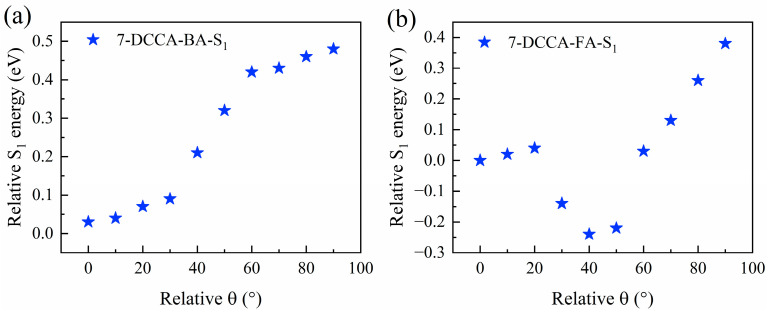
The potential energy surface of the hydrogen bond complex formed by 7-DCCA in BA (**a**) and FA (**b**) solvents at the S_1_ state (Relative θ = 0° refers to the optimized dihedral angle in the ground state).

Figure 7 displays the optimized geometries of the ground (S_0_) and first excited (S_1_) states of 7-DCCA hydrogen-bonded complexes, calculated at the B3LYP/Def2TZVP level. The corresponding bond lengths are summarized in Table S1. The results indicate that in the S_1_ state, the intermolecular hydrogen bonds (C_1_-O_1_···H_1_ and C_3_-O_2_···H_2_) in all four 7-DCCA hydrogen-bonded complexes are significantly shorter than in the S_0_ state. The formation of hydrogen bonds in the excited state leads to an increase in the C_1_-O_1_ and C_3_-O_2_ bond lengths, and the shortening of the hydrogen bonds in the S_1_ state suggests an increase in their strength, which promotes the ICT process. To further reveal the charge transfer characteristics upon photoexcitation, the frontier molecular orbitals (FMOs) of 7-DCCA in different solvents were calculated, as shown in Figure 8. During the HOMO → LUMO transition, the electron density shifts from the diethylamino donor moiety to the coumarin acceptor core, characteristic of π → π* transition [40,41]. The energy gaps (ΔE) of 7-DCCA in different solvents (Diox, BA, FA, and DMSO) are −3.5372 eV, −3.4183 eV, −3.3949 eV, and −3.4455 eV, respectively. Evidently, the energy gap gradually decreases with increasing solvent polarity. The narrowing of the energy gap reduces the energy required for electrons to transition from the ground state to the excited state, thereby promoting the formation of the TICT state [42,43]. The redistribution of electron density primarily occurs within the 7-DCCA framework rather than on the solvent molecules, indicating that solvents modulate the TICT process mainly through hydrogen-bonding and polarization effects. In addition, we also performed a potential energy surface (PES) analysis of the diethylamino dihedral angle in the 7-DCCA hydrogen-bonded complex (Figure 7). The calculation results were obtained at the CAM-B3LYP/Def2TZVP/D3(BJ)/SMD level [44], as shown in Figure 9. The x-axis represents the relative dihedral angle of the diethylamino group in the 7-DCCA hydrogen-bonded complex, and the y-axis represents the relative energy of the S_1_ state hydrogen-bonded complex. The results show that as the relative dihedral angle increases from 0° to 90°, the relative energy of the S_1_ state in the 7-DCCA-BA hydrogen-bonded complex gradually increases, reaching 0.48 eV. In the 7-DCCA-FA hydrogen-bonded complex, when the relative dihedral angle increases to 20°, the energy reaches 0.04 eV, then starts to decrease, reaching the minimum value at 40°. This further indicates that the structural characteristics of the 7-DCCA-FA complex favor the formation of the TICT process, while the 7-DCCA-BA complex, despite its strong hydrogen-donating ability, does not undergo the TICT process due to its weaker polarity. The computational results provide valuable mechanistic insights that are qualitatively consistent with our experimental observations. While the core conclusions of this study are grounded in experimental photophysics, the DFT/TDDFT calculations offer a coherent physical picture that helps interpret the observed solvent-dependent trends. Despite known limitations of the B3LYP functional for charge-transfer states, the calculations successfully capture the qualitative mechanisms underlying the experimental behavior, which suffices for this interpretative study.

AIM (Atoms in Molecules) theory is an important tool for analyzing weak interactions. In AIM theory, the critical points include nuclear positions, bond critical points (BCP), and ring critical points, with BCP being the key point for analyzing weak interactions. By analyzing the properties of the BCP, the strength of intermolecular interactions can be obtained. We used the AIM program to further investigate the electronic density topological structure of the 7-DCCA-solvent complexes (as shown in Figure S2) and calculated the electronic density at the excited-state BCP and the hydrogen bond binding energies. The larger the electronic density, the stronger the chemical bond, and the more negative the binding energy, the stronger the hydrogen bond. According to the bond critical point data we calculated (see Table 7), it can be seen that 7-DCCA has a higher electronic density in FA and DMSO, which allows for the formation of stronger hydrogen bonds. On the other hand, 7-DCCA exhibits stronger hydrogen bond binding energies in FA and DMSO, further confirming that 7-DCCA can form stable hydrogen bonds in these two solvents [45,46,47]. Overall, the theoretical results corroborate the experimental observations and underscore the crucial role of hydrogen bonding in governing the solvent-dependent excited-state charge-transfer dynamics of 7-DCCA.

### 2.5. Excited-State Deactivation Mechanism of 7-DCCA Hydrogen-Bonded Complexes

**Figure 10:**
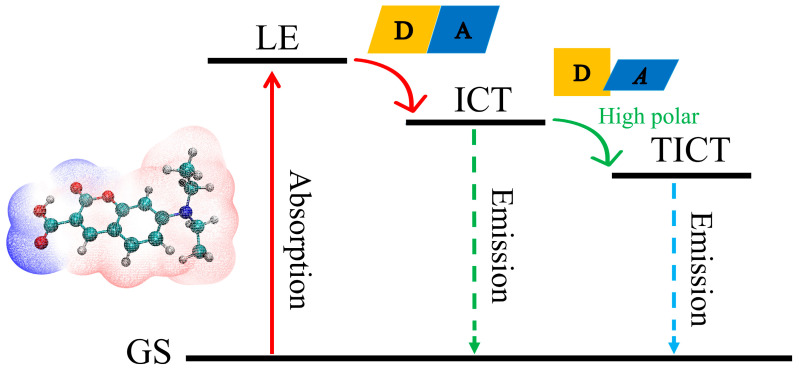
Excited-state deactivation mechanism of 7-DCCA hydrogen-bonded complexes.

Based on the analysis of steady-state and transient spectra, a schematic of the excited-state deactivation mechanism of 7-DCCA in both nonpolar and polar solvents is proposed (Figure 10). In low-polarity solvents like Diox and BA, 7-DCCA rapidly transitions from the ground state to the LE state upon photoexcitation. Within a few hundred femtoseconds, it swiftly converts from the LE state to the ICT state, followed by a solvent relaxation process occurring over tens of picoseconds before ultimately returning to the ground state. Conversely, in highly polar solvents like FA and DMSO, 7-DCCA, upon reaching the ICT state, undergoes an additional configurational evolution pathway known as the TICT process. As solvent polarity increases and intermolecular hydrogen bonding intensifies, the ICT is further enhanced, resulting in a reduction in electron cloud density on the diethylamino donor. This induces a torsional distortion relative to the coumarin backbone, thereby stabilizing the TICT state.

## 3. Materials and Methods

### 3.1. Materials

7-DCCA and solvents, including BA and FA, were purchased from Aladdin (Shanghai, China), while Diox and DMSO were supplied by Macklin (Shanghai, China). All chemicals were used without further purification, and the solvents were of spectroscopic grade. In all experiments, 7-DCCA was dissolved in each solvent at a concentration of 80 μΜ/L, and the solutions were placed in 10 mm diameter quartz cuvettes. All measurements were conducted at room temperature (298 K).

### 3.2. Experimental Methods

Steady-state absorption and fluorescence spectra were recorded using a FluoroMax+ fluorescence spectrometer from HORIBA (Kyoto, Japan). Femtosecond transient absorption spectra (fs-TA) were acquired using a femtosecond pump-probe setup consisting of an Astrella femtosecond laser (Coherent, Santa Clara, CA, USA), a TOPAS-Prime optical parametric amplifier (Coherent), and a Transpec FS pump-probe system (Zhongzhi Keyi, Beijing, China). The 800 nm pulses (1 kHz, 35 fs) generated by the femtosecond laser were split into two beams. The stronger pulse was directed through the optical parametric amplifier to generate pump pulses, while the weaker pulse was passed through an optical delay line (0–4 ns) and a white crystal to produce a white-light continuum probe. The pump and probe beams were spatially overlapped at the sample position, and the resulting signal was detected, allowing for the acquisition of transient absorption spectra. The excitation wavelength used in the experiments was set to 375 nm [48].

### 3.3. Computational Methods

Ground-state geometries of 7-DCCA and its hydrogen-bonded complexes were optimized using DFT, and their excited-state structures, vertical excitation energies, fluorescence emission energies, and oscillator strengths were calculated using TDDFT. All calculations were performed at the B3LYP/Def2TZVP level with Grimme’s D3 dispersion correction and Becke–Johnson damping (D3BJ) to capture weak intermolecular interactions, and the SMD implicit solvent model was employed to account for solvent effects in our qualitative mechanistic model. In addition, excited-state potential energy surface (PES) scanning was performed at the CAM-B3LYP/Def2TZVP level. ESP maps and FMOs were generated using the Multiwfn 3.8 program to visualize charge distributions and analyze their roles in intramolecular charge transfer [49,50]. We also used AIM analysis to study the electronic density topological structure of the 7-DCCA hydrogen-bonded complex. This analysis allows for the identification of critical points and the study of chemical bonds, including weak non-covalent interactions. All quantum calculations were carried out using the Gaussian 09 software package [51].

## 4. Conclusions

By combining steady-state and transient absorption spectroscopy with quantum chemical calculations, we have elucidated the excited-state dynamics and underlying molecular mechanisms of 7-DCCA in diverse solvent environments. The results demonstrate that solvent polarity and hydrogen-bonding interactions are pivotal in regulating the TICT of solvent–solute hydrogen-bond complexes. Analyses employing the Kamlet–Taft and Catalán 4P models reveal that the photophysical behavior of 7-DCCA is not solely dictated by solvent polarity but also by the cooperative effects of hydrogen-bond donation and acceptance. Steady-state emission spectra exhibit a marked red shift with increasing solvent polarity, reflecting a stronger propensity for the transition from the LE to the TICT state. The fitting parameters derived from fs-TA further indicate that this transformation occurs on a sub–10 ps timescale, indicating that intermolecular hydrogen bonding efficiently stabilizes the transient state and facilitates charge redistribution. Theoretical calculations support these observations, confirming that highly polar solvents with strong hydrogen-bonding ability affect the energy barrier of the ICT → TICT transition to some extent by forming stable intermolecular hydrogen bonds. Together, these results provide molecular-level insight into the fundamental photophysics of 7-DCCA and establish a guiding principle for suppressing TICT formation and enhancing fluorescence efficiency through precise control of solvent polarity and hydrogen-bond interactions. The elucidated solvent–solute coupling mechanism has broad implications for the rational design of high-brightness fluorescent probes and stable organic optoelectronic materials with tunable photoactive properties.

## Figures and Tables

**Table 1:** K-T (*α*, *β*, π*) and Catalán (*SA*, *SB*, *SP*, *SdP*) parameters for different solvents.

Solvent	*α*	*β*	π*	*SA*	*SB*	*SP*	*SdP*
1,4-dioxane	0.00	0.37	0.55	0.000	0.444	0.737	0.312
Butanol	0.84	0.84	0.47	0.341	0.809	0.674	0.655
Formamide	0.71	0.48	0.97	0.549	0.414	0.814	1.006
Dimethyl sulfoxide	0.00	0.76	1.00	0.072	0.647	0.830	1.000
Acetonitrile	0.19	0.40	0.75	0.044	0.286	0.645	0.974
Tetrahydrofuran	0.00	0.55	0.58	0.000	0.591	0.714	0.634
*N*,*N*-dimethyIformamide	0.00	0.69	0.88	0.031	0.613	0.977	0.759
Ethanol	0.86	0.75	0.54	0.400	0.658	0.633	0.783
Methanol	0.98	0.66	0.60	0.605	0.545	0.608	0.904
Ethyl acetate	0.00	0.45	0.55	0.000	0.542	0.656	0.603
Acetic acid	1.12	0.45	0.64	0.689	0.390	0.651	0.676
dichloromethane	0.13	0.10	0.82	0.040	0.178	0.761	0.769
H_2_O	1.17	0.47	1.09	1.062	0.025	0.681	0.997
chloroform	0.20	0.10	0.58	0.047	0.071	0.783	0.614
2-propanol	0.76	0.84	0.48	0.283	0.830	0.633	0.808
Cyclohexane	0.00	0.00	0.00	0.000	0.073	0.683	0.000

**Table 2:** Absorption and emission peaks (eV), and Stokes shifts (cm^−1^) of 7-DCCA in different solvents.

Solvent	Abs	Em	Stokes Shift
1,4-dioxane	2.952	2.695	2017
Butanol	2.924	2.689	1893
Formamide	3.046	2.638	3293
Dimethyl sulfoxide	2.938	2.644	2375
Acetonitrile	2.945	2.666	2070
Tetrahydrofuran	2.890	2.684	1665
*N*,*N*-dimethyIformamide	2.897	2.610	2313
Ethanol	2.960	2.678	2268
Methanol	2.986	2.689	2404
Ethyl acetate	2.910	2.683	1828
Acetic acid	3.031	2.701	2663
dichloromethane	2.980	2.661	2580
H_2_O	3.084	2.588	3999
chloroform	2.959	2.672	2315
2-propanol	2.924	2.713	1703
Cyclohexane	2.966	2.850	934

**Table 3:** Statistical best-fit parameters (×10^3^ cm^−1^) of the empirical solvatochromic model for 7-DCCA.

	Kamlet-Taft Model					Catalán 4P Model					
	*ν*_0_	*a*	*b*	*s*	R^2^	*ν*_0_	*C_SA_*	*C_SB_*	*C_SP_*	*C_SdP_*	R^2^
Abs	23.67	0.80	−1.15	0.67	0.875	23.90	1.01	−0.84	0.11	0.04	0.881
Em	22.75	0.02	−0.26	−1.55	0.881	23.01	−0.23	0.35	−1.71	−0.48	0.922
Stokes shift	0.91	0.81	−0.88	2.20	0.961	0.87	1.31	−1.14	1.88	0.40	0.958

**Table 4:** The calculated vertical excitation energies (eV) and corresponding oscillator strengths of 7-DCCA hydrogen-bonded complexes.

Molecule	7-DCCA-Diox	7-DCCA-BA	7-DCCA-FA	7-DCCA-DMSO
S_1_	3.06 (0.744) H → L98.4%	3.01 (0.760) H → L98.6%	3.08 (0.845) H → L98.4%	3.05 (0.791) H → L98.4%
S_2_	3.42 (0.000)	3.56 (0.002)	3.75 (0.004)	3.23 (0.000)
S_3_	3.84 (0.000)	4.10 (0.002)	4.33 (0.000)	3.26 (0.000)
S_4_	4.12 (0.007)	4.11 (0.000)	4.38 (0.038)	4.1.0 (0.004)
S_5_	4.16 (0.000)	4.41 (0.167)	4.39 (0.138)	4.22 (0.000)
S_6_	4.48 (0.123)	4.66 (0.000)	4.56 (0.001)	4.26 (0.000)

**Table 5:** The calculated fluorescence emission energies (eV) and corresponding oscillator strengths of 7-DCCA hydrogen-bonded complexes.

Molecule	7-DCCA-Diox	7-DCCA-BA	7-DCCA-FA	7-DCCA-DMSO
S_1_	2.78 (0.446) H → L99.1%	2.77 (0.916) H → L99.1%	2.74 (0.999) H → L99.1%	2.76 (0.924) H → L99.2%
S_2_	3.04 (0.000)	3.85 (0.015)	3.85 (0.021)	3.04 (0.000)
S_3_	3.51 (0.004)	3.90 (0.064)	4.15 (0.000)	3.06 (0.000)

**Table 6:** Kinetic parameters obtained from fitting for 7-DCCA in Diox (**a**), BA (**b**), FA (**c**), and DMSO (**d**).

Sample	Solvent	τ_1_/ps	τ_2_/ps	τ_3_/ps	τ_4_
7-DCCA	Diox (**a**)	0.43 ± 0.01	—	69.77 ± 1.21	2806.31 ± 82.63
	BA (**b**)	0.45 ± 0.01	—	25.08 ± 0.84	418.35 ± 6.57
	FA (**c**)	0.40 ± 0.02	5.51 ± 0.32	154.05 ± 18.59	730.33 ± 17.31
	DMSO (**d**)	0.22 ± 0.01	3.01 ± 0.51	41.45 ± 6.57	216.73 ± 2.21

**Table 7:** The electronic density (ρ) at the bond critical point and the corresponding hydrogen bond energy (ΔE).

	BCP	ρ(r_BCP_) (au)	ΔE (kcal/mol)
7DCCA-Diox	C_1_-O_1_···H_1_	0.0061	−0.6185
	C_3_-O_2_···H_2_	0.0052	−0.4177
7DCCA-BA	C_1_-O_1_···H_1_	0.2585	−56.9239
	C_3_-O_2_···H_2_	0.2741	−60.4039
7DCCA-FA	C_1_-O_1_···H_1_	0.3191	−70.4425
	C_3_-O_2_···H_2_	0.2815	−62.0547
7DCCA-DMSO	C_1_-O_1_···H_1_	0.2708	−59.6678
	C_3_-O_2_···H_2_	0.2717	−59.8685

## Data Availability

All data supporting the findings of this study will be made available by the authors on request.

## Supplementary Materials

The following supporting information can be downloaded at: https://www.mdpi.com/article/10.3390/molecules31010076/s1, Figure S1. The electrostatic potential maps of 7-DCCA in the S0 state and S1 state in Diox (a)/(e), BA (b)/(f), FA (c)/(g), and DMSO (d)/(h). Figure S2. AIM topological structures of the excited-state hydrogen-bonded complexes of 7-DCCA with Diox (a), BA (b), FA (c), and DMSO (d). Table S1. Key bond length (Å) information for 7-DCCA hydrogen-bonded complexes.
