# Supplementary material for: Solvent-Mediated Control of Twisted Intramolecular Charge Transfer in 7-(Diethylamino)coumarin-3-carboxylic Acid
Source: Molecules. 2025 Dec 24;31(1):76. doi: 10.3390/molecules31010076 (PMC12787091; doi:10.3390/molecules31010076)
Supplement: Supplementary file 1 [file molecules-31-00076-s001.zip › molecules-3996571-supplementary.pdf]

Supplementary materials

Solvent-Mediated Control of Twisted Intramolecular Charge Transfer in 7-(Diethylamino) coumarin-3-carboxylic Acid

Xilin Bai [1,2,*], Jing Xiao [1], Bingqi Du [1], Duidui Liu [1], Yanzhuo Wang [1], Shujing Shi [1] and Jing Ge [1,*]

1. School of Physics and Electronic Engineering, Shanxi Normal University, No. 339, Taiyu Road, Taiyuan, 030031, China;

2. Department of Chemical Physics, University of Science and Technology of China, No. 96, Jinzhai Road, Hefei, 230026, China;

* Correspondence: bxl5630@mail.ustc.edu.cn (X.B.); 703366@sxnu.edu.cn (J.G.)

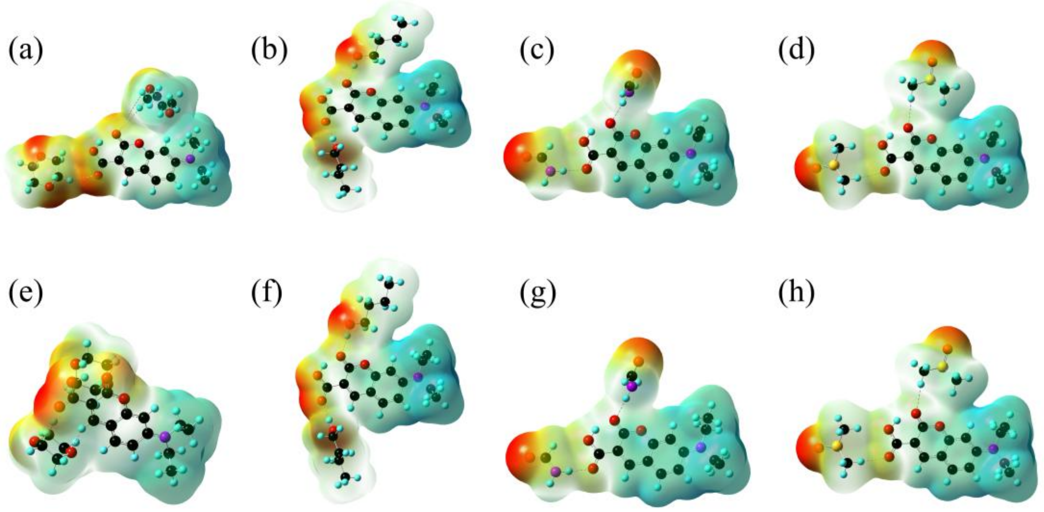

Figure S1. The electrostatic potential maps of 7-DCCA in the S_0 state and S_1 state in Diox (**a**)/(**e**), BA (**b**)/(**f**), FA (**c**)/(**g**), and DMSO (**d**)/(**h**).

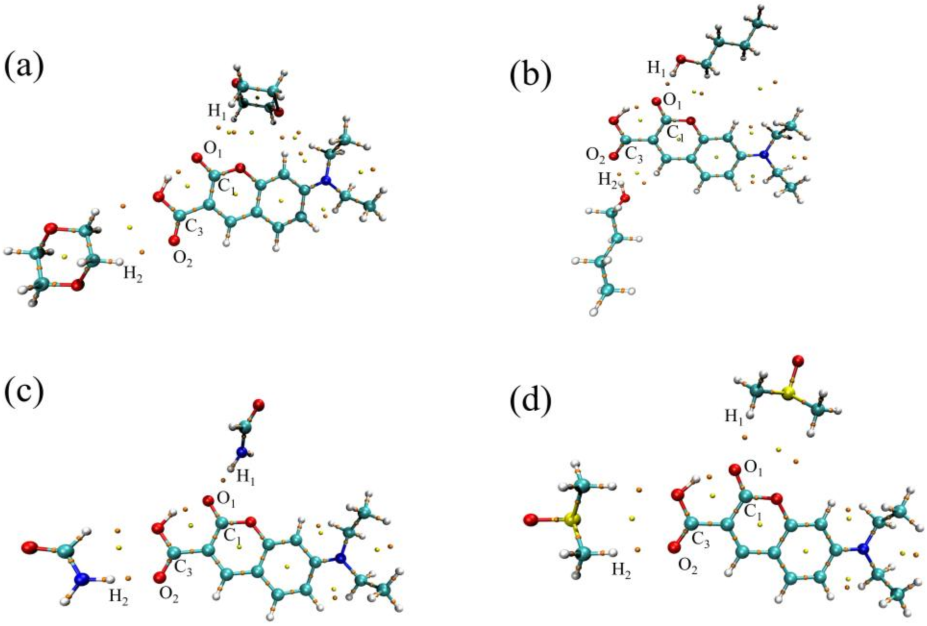

Figure S2. AIM topological structures of the excited-state hydrogen-bonded complexes of 7-DCCA with Diox (**a**), BA (**b**), FA (**c**), and DMSO (**d**).

Table S1. Key bond length (Å) information for 7-DCCA hydrogen-bonded complexes.

Molecule	7DCCA-Diox		7DCCA-BA		7DCCA-FA		7DCCA-DMSO	
	S_0	S_1	S_0	S_1	S_0	S_1	S_0	S_1
C_1-O_1	1.225	1.235	1.236	1.245	1.237	1.245	1.231	1.239
C_3-O_2	1.213	1.217	1.225	1.231	1.226	1.233	1.216	1.222
C_1-O_1···H_1	3.257	3.230	1.868	1.848	1.949	1.932	2.472	2.416
C_3-O_2···H_2	2.542	2.358	1.796	1.778	1.923	1.902	2.380	2.343
